# Circuit Mechanisms of Neurodegenerative Diseases: A New Frontier With Miniature Fluorescence Microscopy

**DOI:** 10.3389/fnins.2019.01174

**Published:** 2019-10-31

**Authors:** Craig T. Werner, Christopher J. Williams, Mercedes R. Fermelia, Da-Ting Lin, Yun Li

**Affiliations:** ^1^Intramural Research Program, National Institute on Drug Abuse, National Institutes of Health, Baltimore, MD, United States; ^2^Department of Zoology and Physiology, University of Wyoming, Laramie, WY, United States; ^3^The Solomon H. Snyder Department of Neuroscience, Johns Hopkins School of Medicine, Johns Hopkins University, Baltimore, MD, United States

**Keywords:** neurodegenerative disorders, miniature fluorescence microscopy, miniscope, *in vivo* calcium imaging, deep brain imaging, longitudinal recording

## Abstract

Neurodegenerative diseases (NDDs), such as Alzheimer’s disease (AD), Parkinson’s disease (PD), Huntington’s disease (HD), amyotrophic lateral sclerosis (ALS), and frontotemporal dementia (FTD), are devastating age-associated brain disorders. Significant efforts have been made to uncover the molecular and cellular pathogenic mechanisms that underlie NDDs. However, our understanding of the neural circuit mechanisms that mediate NDDs and associated symptomatic features have been hindered by technological limitations. Our inability to identify and track individual neurons longitudinally in subcortical brain regions that are preferentially targeted in NDDs has left gaping holes in our knowledge of NDDs. Recent development and advancement of the miniature fluorescence microscope (miniscope) has opened up new avenues for examining spatially and temporally coordinated activity from hundreds of cells in deep brain structures in freely moving rodents. In the present mini-review, we examine the capabilities of current and future miniscope tools and discuss the innovative applications of miniscope imaging techniques that can push the boundaries of our understanding of neural circuit mechanisms of NDDs into new territories.

## Introduction

Neurodegenerative diseases (NDDs), such as Alzheimer’s disease (AD), Parkinson’s disease (PD), Huntington’s disease (HD), amyotrophic lateral sclerosis (ALS), and frontotemporal dementia (FTD), are prevalent and incredibly devastating diseases, with an estimated 6 million people affected by NDDs in the United States alone. A common risk factor for most NDDs is advancing age, and with a globally growing elderly population, the number of cases is expected to increase rapidly worldwide in the coming years. Substantial efforts have been undertaken to study NDDs, which have led to fundamental insights into these diseases. Still, the neurobiology that underlies NDDs is not well understood. With limited knowledge, therapeutic interventions have been developed to delay deterioration and provide temporary relief of symptoms, but there remains a tremendous need to improve treatments.

Although NDDs uniquely affect specific neuronal subpopulations, the accumulation of distinct protein-based macroscopic deposits that cause neuronal loss is a common hallmark of NDDs. In parallel with progressive neuronal loss, synaptic dysfunction, altered calcium homeostasis, aberrant neural activity, abnormal neuronal plasticity, and impairment in large-scale neural circuits are well-documented features of many NDDs (Palop and Mucke, 2016; Frere and Slutsky, 2018; Pchitskaya et al., 2018; Jackson et al., 2019). The interdependence between abnormal protein depositions, neuronal loss, and dysfunctional neural networks remains to be determined. Nevertheless, growing evidence suggests that many neurological deficits in NDDs may reflect functional impairment in neural circuits rather than neuronal loss (Palop et al., 2006; Seeley et al., 2009), suggesting that targeting reversible neural circuits holds therapeutic potential and deserves substantial consideration (Laxton et al., 2010; Bakker et al., 2012; Canter et al., 2016).

**FIGURE 1:**
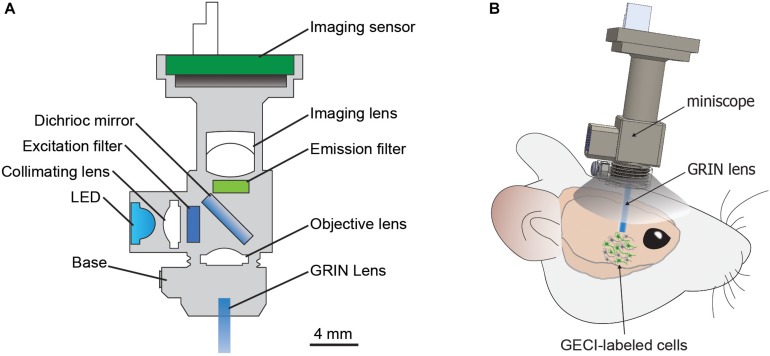
The miniscope. **(A)** Basic components of miniScope and GRIN lens system. **(B)** Schematic for a mounted miniScope with the GRIN lens aimed at GECI-labeled cells in a deep brain structure. GRIN, gradient index lens; GECI, genetically encoded calcium indicator.

Due to technical challenges in performing longitudinal studies on individual neurons in large-scale populations from subcortical brain regions in freely moving animals, our current understanding of the neural circuit mechanisms that underlie NDDs and associated symptoms remains limited. In the present mini-review, we focus on the potential of the miniature fluorescence microscope (miniscope), a recently developed *in vivo* calcium imaging technology (Figure 1; Aharoni and Hoogland, 2019), to provide new insights into dysfunctional neural circuits that contribute to the pathogeneses of NDDs. We first discuss a brief history of the miniscope, and then describe specific capabilities of the miniscope with potential applications to study microcircuit dysfunction in models of NDDs.

## A Brief History of the Miniscope

The observation of *in vivo* calcium transients using microscopy would not be possible without calcium indicators (Grienberger and Konnerth, 2012). Genetically encoded calcium indicators (GECIs; Miyawaki et al., 1997), and GCaMP-family GECIs in particular (Nakai et al., 2001; Tian et al., 2009; Chen et al., 2013), have proved to be incredibly important tools, and have played important roles in defining what can be achieved with calcium imaging. Ongoing advancements in calcium sensors continue to push beyond current limitations, and with activity sensors also moving beyond calcium, voltage, neurotransmitter, and ion sensors will help to shape a very different future for fluorescence microscopy (Table 1; Lin and Schnitzer, 2016; Deo and Lavis, 2018).

**TABLE 1:** Selected genetically encoded indicators.

**Type**	**Sensor examples**	**Reference**
Calcium	GCaMP6	Chen et al., 2013
	GCaMP7	Dana et al., 2019
	jRGECO1	Dana et al., 2016
	XCaMPs	Inoue et al., 2019
Voltage	QuasAr2	Hochbaum et al., 2014
	ArcLight	Kwon et al., 2017
Dopamine	GRAB_DA_	Sun et al., 2018
	dLight1	Patriarchi et al., 2018
Glutamate	SF-iGluSnFR	Marvin et al., 2018
Acetylcholine	GACh	Jing et al., 2018
Potassium	KIRIN1	Shen et al., 2019

Coinciding with advancements in fluorescent activity reporters has been progress in imaging instrumentation. Since the development of two-photon microscopy (Denk et al., 1990), studies examining calcium activity have increased dramatically. Imaging cortical activity through cranial windows in head-fixed animals using a variety of behaviors has provided many new insights into the neuronal encoding of behavior (Yang et al., 2009; Komiyama et al., 2010), and the emergence of Gradient Index (GRIN) lenses, which relay images from one end to the other to provide an optical interface to the brain, has allowed for endoscopic imaging of deep brain regions (Barretto et al., 2009; Yang et al., 2019; Zhang et al., 2019). Examination of neuronal activity during behavior in freely moving animals was then made possible by the development of fiber-optic based microscopes (Helmchen et al., 2001, 2013; Flusberg et al., 2008; Sawinski et al., 2009) and miniature fluorescence microscopes with two-photon (Helmchen et al., 2001; Sawinski et al., 2009; Liang et al., 2017; Zong et al., 2017) or epifluorescent light sources (Ghosh et al., 2011).

The first miniscope was developed by Ghosh et al. (2011), which included a number of unique features. The miniscope was head-mounted with fluorescence excitation and detection onboard rather than away from the microscope, and also bypassed cost limitations of table-top setups by using commercially available and inexpensive technologies. The miniscope offers many unique advantages, including the ability to perform recordings that are cell-type specific, large-scale (hundreds of cells can be recorded), and longitudinal in nature [cells can be recorded for months (Zhang et al., 2019)]. These features provide a unique tool to study NDDs, as the miniscope can image cells in microcircuits during the development of NDD-like pathophysiology and behavior in animal models.

## *In Vivo* Deep Brain Imaging in Freely Moving Animals

Many behavioral manifestations of NDDs have been modeled in animals (Gitler et al., 2017), and understanding the microcircuitry that encodes disease-related aberrant behaviors could provide essential insights into the neurobiology underlying NDDs. However, observing neuronal activity during behaviors in freely moving animals has been technically challenging. Two-photon imaging using table-top setup approaches requires head fixation, which limits the usability to certain behavioral assays. Moreover, differences in neural activity have been reported in head-fixed animals compared to freely moving animals, suggesting that head-restrained and natural conditions have different neural correlates (Ravassard et al., 2013; Aghajan et al., 2015). Various instruments have been developed to examine neuronal activity during behavior in freely moving animals (Helmchen et al., 2001, 2013; Flusberg et al., 2008; Sawinski et al., 2009; Liang et al., 2017; Zong et al., 2017), but the miniscope offers a unique option that is accessible, compact, and affordable (Figure 1 and Table 2; Aharoni and Hoogland, 2019).

**TABLE 2:** Open-source miniscopes.

**Miniscope**	**Developer**	**Platform**	**Reference**
UCLA miniscope	UCLA	http://miniscope.org/index.php/Main_Page	Cai et al., 2016
FinchScope	Boston University	https://github.com/gardner-lab/FinchScope	Liberti et al., 2016
miniScope	NIDA	https://github.com/giovannibarbera/miniscope_v1.0	Barbera et al., 2016
CHEndoscope	University of Toronto	https://github.com/jf-lab/chendoscope	Jacob et al., 2018
cScope	Princeton	https://github.com/PrincetonUniversity/cScope	Scott et al., 2018

An additional technical challenge is accessing deep brain structures that are preferentially affected in many NDDs. Until recently, the ability of fluorescence microscopy to monitor neuron activity at subcellular resolution has been limited to superficial depths of ∼1 mm (Mittmann et al., 2011). This is due to light scattering that occurs in tissue (Ji, 2014), as well as tissue damage and inflammatory responses that occurs when removing overlaying tissue to provide optical access to a structure of interest. Imaging neurons in deeper brain structures is more practical with endoscopic techniques, which include insertion of lenses into the brain. The use of smaller-diameter GRIN lenses can reduce tissue damage (Bocarsly et al., 2015; Yang et al., 2019) and minimize the effect on behavior (Lee et al., 2016), which can be further improved with the use of robotic surgical instruments (Liang et al., 2019).

## Longitudinal Tracking of Individual Cells to Study Ndds

Aging is a leading risk factor for many NDDs. While each NDD demonstrates unique spatiotemporal patterns of degeneration, the often-slow advancement of these diseases provides a similarly distinct challenge for researchers to study. A major cause of age-related degeneration is the accumulation of disease-specific misfolded proteins that lead to progressive loss of neuronal function (Hung et al., 2010; Chiti and Dobson, 2017). However, changes in patterned neural and circuit activity have also been observed in most NDDs and are believed to contribute to the onset and progression of disease states (Palop et al., 2006; Seeley et al., 2009).

### Hyperexcitability in Early Stage of NDDs

Hyperexcitability has been observed in a number of NDDs. Cortical and peripheral hyperexcitability has been well-documented in clinical studies of ALS patients (Mills and Nithi, 1997; Vucic and Kiernan, 2006; Vucic et al., 2008; Williams et al., 2013; Menon et al., 2015), and is among the earliest pathologies identified in ALS patients (Bae et al., 2013) and during early stages of ALS-like pathology in rodent models (van Zundert et al., 2008; Zhang et al., 2016; Marcuzzo et al., 2019). Therefore, it is presumed that neuronal hyperexcitability is an important factor that leads to neuronal loss in ALS. In AD, although hypoactivity is a key feature of late stages of the disease (Braak and Braak, 1991; Nestor et al., 2003), paradoxical hyperactivity has been observed in multiple brain regions of AD patients during pre-symptomatic stages (Sperling, 2007; Dickerson and Sperling, 2008; Miller et al., 2008; Pihlajamaki et al., 2009; Yassa et al., 2010). Furthermore, chronic aberrant increases in excitatory neuronal activity have been detected in the hippocampal and cortical regions in AD mouse models (Palop et al., 2007; Busche et al., 2008), and increasing neural activity has been demonstrated to directly promote the production and secretion of amyloid-β (Aβ) peptide (Cirrito et al., 2005, 2008; Bero et al., 2011), whose excessive accumulation appears to play a causal role in AD (Stargardt et al., 2015). However, how hyperexcitable neurons encode behavior and contribute to disease progression remains unknown.

### Therapeutic Potential of Targeting Hyperactivity

Targeting hyperactivity in mouse models of AD and ALS has been demonstrated to improve signatures of neural injury (Sanchez et al., 2012; Yuan and Grutzendler, 2016; Zhang et al., 2016; Haberman et al., 2017), and, importantly, clinical trials aiming to reduce hippocampal hyperactivity showed positive effects in patients with amnestic cognitive impairment (Bakker et al., 2012). The most widely prescribed drug for ALS, riluzole, reduces excitatory synaptic activity in the brain (Doble, 1996), and memantine, a therapeutic drug approved for use in late-stage AD, functions as a NMDA receptor antagonist to counteract hyperactive glutamatergic circuits (Zhu et al., 2013). But still, there remains a need to determine how these drugs affect neuronal activity to provide therapeutic relief to disease-related behavioral symptoms. Examining the activity of individual neurons across time could provide insights into how microcircuits encode pathophysiological behaviors as diseases advance. The miniscope is able to track individual neurons for several months during behaviors in freely moving animals (Liang et al., 2018; Zhang et al., 2019), providing an opportunity to examine how changes in activity in individual neurons within a microcircuit across long periods of time affect behavior.

## Imaging Specific Cellular Subtypes to Study Ndds

Each NDD has a signature pattern of degeneration progression. Particular circuits are vulnerable, and specific subtypes of cells within circuit-associated brain regions are targeted. In combination with transgenic rodent lines and/or cell-type specific and inducible promotors for vectors in gene therapy, specific neuron subtypes, and other cell-types in the brain can be imaged with the miniscope. This capability provides exciting opportunities for new insights into how neuronal subtypes encode pathology-related behaviors in models of NDDs.

### Imaging Dopaminergic, Cholinergic, and Medium Spiny Neurons

The manifestation of PD symptoms is believed to be the cause of dopaminergic neuron loss in the substantia nigra pars compacta (SNc) that leads to an imbalance in basal ganglia direct and indirect pathways (McGregor and Nelson, 2019). Studies with parkinsonian rodents have linked activity changes to discrete neuronal populations with potentially distinct contributions to and/or alleviation of parkinsonian motor symptoms (Mallet et al., 2016; Parker et al., 2018; Ryan et al., 2018; Sagot et al., 2018). Using transgenic Cre-driver mouse lines and a commercial miniscope, Parker et al. (2018) reported that medium spiny neurons (MSNs) in the direct and indirect pathways displayed bidirectional changes in firing rate in parkinsonian mice. Firing rates in MSNs, along with behavioral abnormalities, were reversed by administration of L-DOPA or a dopamine receptor D_2_ receptor agonist (Parker et al., 2018). While this study demonstrated some of the capabilities of miniscope imaging, numerous questions still remain related to firing rate, pattern, and synchronization of neurons in the SNc and other brain regions that are believed to contribute to PD pathology.

Selective vulnerability of MSNs in the basal ganglia is a characteristic of HD (Ross and Tabrizi, 2011). MSNs are unique in that they not only receive dopaminergic input from the midbrain but also glutamatergic input from the cortex and other brain regions. Direct imaging of MSN activity in the basal ganglia would provide useful clues to determine intrinsic properties of MSNs that may lead to vulnerability in HD.

Neuromodulatory system dysfunction has been linked to a number of NDDs, but how specific neuromodulatory systems encode behavior in models of NDD has yet to be examined. Selective loss of cholinergic neurons have been observed in postmortem brains of AD patients (Whitehouse et al., 1981; Mesulam et al., 2004), and reductions in cholinergic markers are strongly correlated with cognitive decline in PD patients (Perry et al., 1985). Importantly, clinical studies support beneficial effects of cholinesterase inhibitor treatment in both PD and AD patients (Emre et al., 2004; Zhu et al., 2013), suggesting a need to better understand how cholinergic neuron dysfunction contributes to symptoms in PD, AD, and other NDDs.

### Imaging Specific Inhibitory Interneurons

γ-aminobutyric acid (GABA) plays a central role in regulating neuronal excitability and maintaining balanced network activity, and inhibitory interneuron abnormalities also contribute to a number of NDDs. Inhibitory interneuron dysfunction has been suggested to contribute to network hypersynchrony (Palop and Mucke, 2016) that underlies frequent epileptic activity that occurs in AD patients (Vossel et al., 2016, 2017), as deficits in inhibitory interneurons result in impaired oscillatory rhythm and network hyperactivity in AD mouse models (Baglietto-Vargas et al., 2010; Villette et al., 2010; Verret et al., 2012; Hamm et al., 2017). In addition, reductions of subpopulations of inhibitory interneurons have been described in AD mouse models and postmortem brains of AD patients (Ramos et al., 2006; Takahashi et al., 2010; Albuquerque et al., 2015; Mahar et al., 2016). Dysfunctional interneurons have also been linked to upper motor neuron hyperexcitability in an ALS/FTD model (Zhang et al., 2016). Imbalance of excitation-to-inhibition (E/I) due to interneuron impairment has been suggested to be a potential common driver for AD and ALS, as well as other NDDs (Do-Ha et al., 2018; Ambrad Giovannetti and Fuhrmann, 2019). Studies on inhibitory interneurons using miniscope imaging in NDD models could shed light on this theory and provide information on inhibitory interneurons in NDD pathogenesis.

### Imaging Specific Glial Cells

A variety of glial cells have complex reciprocal interactions with neurons to support their functions and are active participants in neural circuit refinement (Paolicelli et al., 2011; Schafer et al., 2012; Risher et al., 2014; Tani et al., 2014). For instance, subsets of astrocytes in the basal ganglia release glutamate to selectively activate homotypic synapses to regulate neural networks (Martin et al., 2015). Dysfunctional glial cells contribute to the disease progression in both HD and ALS through non-cell-autonomous toxicity (Yamanaka et al., 2008; Haidet-Phillips et al., 2011; Creus-Muncunill and Ehrlich, 2019). Neuroinflammation that results from chronic activation of immune responses are often mediated by microglia and occur in susceptible regions, and are believed to contribute to age-related degeneration (McGeer and McGeer, 2004; Cappellano et al., 2013). Microglial activation and reactive astrogliosis are observed in many NDDs, and reactive astrocytes lose many normal functions and gain new abnormal functions. Synapse elimination by microglial and astrocytes may contribute to the loss of presynaptic terminals and dendritic spines (Chung et al., 2015; Henstridge et al., 2019), one of the earliest features associated with cognitive impairment in many NDDs (Terry et al., 1991; Scheff et al., 2006). Glial cells may selectively remove excitatory or inhibitory synapses to compensate for disease-associated changes at specific brain regions (Hong et al., 2016; Aono et al., 2017; Paolicelli et al., 2017), or may act as a primary driver for the E/I imbalance by directly inducing excessive synaptic pruning (Serrano-Pozo et al., 2013; Rodriguez et al., 2014). Studying glial activity though miniscope calcium imaging might provide useful information on the mechanism of how glial dysfunctions contribute to NDDs.

## New Frontiers in Miniscope Imaging

The current generation of miniscopes offers many exciting possibilities for the study of NDDs. Innovation of the miniscope has been dramatically accelerated by open-source sharing of miniscope projects (Table 2; Aharoni and Hoogland, 2019), with functional improvements that hold the promise of new frontiers. The next generation of miniscopes and fluorescence sensors boast capabilities to distinguish two neuron subtypes simultaneously, manipulate cellular activity while imaging, image cellular events with non-calcium indicators, and record electrical properties (Deo and Lavis, 2018; Aharoni and Hoogland, 2019).

### GECIs With Spectrally Separable Wavelengths

In the case of NDDs, it would be advantageous to simultaneously image a preferentially affected cell-type population, as those described above, while also imaging surrounding cell-types. This would provide information to understand how dysfunction of a vulnerable cellular population not only affects behavior, but also the other cells in the local microcircuit. Development of GECIs (Table 1) with spectrally separable wavelengths (Chen et al., 2013; Dana et al., 2016; Inoue et al., 2019) provide potential to examine the concomitance of two distinct neuronal populations correlated to specific behaviors, which can be accomplished a number of ways, including using a Cre-On/Cre-off approach (Saunders et al., 2012).

GECIs with distinct wavelengths also provide an opportunity to concurrently image and manipulate neural activity. Miniscope imaging offers correlational insight into neural activity that encodes behavior, but manipulating neural activity concurrently with imaging can offer insight into causal roles of cell populations within microcircuits (Stamatakis et al., 2018). This task can be achieved by using light-driven channels that have minimal overlapping spectra with a fluorescent activity sensor. One could also model dysregulated neuronal firing observed in NDDs with optogenetics while recording surrounding neuron activity during behavior.

### Non-calcium Fluorescence Activity Sensors

Fluorescence imaging has focused primarily on measuring intracellular calcium transients. However, florescent sensors for dopamine transients (Patriarchi et al., 2018; Sun et al., 2018), glutamate transients (Marvin et al., 2013, 2018), intracellular potassium ion concentration (Shen et al., 2019) and voltage signals (Lin and Schnitzer, 2016; Deo and Lavis, 2018), among others, have been developed for imaging (Table 1), with more to come. Many NDDs feature a variety of aberrant signaling, including dysfunction of dopamine and glutamate (Heath and Shaw, 2002; Hynd et al., 2004; Lewerenz and Maher, 2015; Schwab et al., 2015; Poewe et al., 2017), which provides an abundance of opportunities to study NDDs with these cutting-edge sensors. For example, dopamine sensors could be used to image dopamine transients in the SNc during progressive loss of dopaminergic neurons.

Extensive evidence suggests that many NDDs may be characterized by alterations in single-cell firing patterns and dyssynchronization between circuit nodes. Electrophysiological techniques currently offer unparalleled temporal resolution, but are unable to record spatiotemporal dynamics of action potential activity in ensembles with single-neuron resolution (Henze et al., 2000; Obien et al., 2014). Fluorescence imaging infers neural activity with high sensitivity but is traditionally unable to accurately resolve individual action potentials during high frequency firing due to slow decays. Engineering of voltage-responsive fluorophores is advancing rapidly with hopes of providing fluorescence responses that can greatly improve temporal resolution (Deo and Lavis, 2018; Quicke et al., 2019).

## Conclusion

Capable of performing cell-type specific recordings on large-scale populations longitudinally, the miniscope offers opportunities to ask new and exciting questions in NDDs. Studies utilizing miniscope imaging are increasing rapidly across disciplines of neuroscience but are not commonly being used in NDD models. It is our hope that this mini-review, while by no means exhaustive, brings attention to the powerful capabilities of miniscope imaging, and offers a glimpse into potential questions that could be answered with miniscope experiments in studies of NDDs.

## Author Contributions

CTW, CJW, MF, D-TL, and YL contributed to the preparation of the manuscript. CTW, D-TL, and YL wrote and edited the manuscript.

## Conflict of Interest

The authors declare that the research was conducted in the absence of any commercial or financial relationships that could be construed as a potential conflict of interest.
